# Activation of respiratory-related bursting in an isolated medullary section from adult bullfrogs

**DOI:** 10.1242/jeb.245951

**Published:** 2023-09-22

**Authors:** Sandy E. Saunders, Joseph M. Santin

**Affiliations:** Biological Sciences, University of Missouri, Columbia, MO 65211, USA

**Keywords:** Bullfrog, Respiratory rhythm generation, Inhibition, Episode formation, Breathing, Control of breathing

## Abstract

Breathing is generated by a rhythmic neural circuit in the brainstem, which contains conserved elements across vertebrate groups. In adult frogs, the ‘lung area’ located in the reticularis parvocellularis is thought to represent the core rhythm generator for breathing. Although this region is necessary for breathing-related motor output, whether it functions as an endogenous oscillator when isolated from other brainstem centers is not clear. Therefore, we generated thick brainstem sections that encompass the lung area to determine whether it can generate breathing-related motor output in a highly reduced preparation. Brainstem sections did not produce activity. However, subsaturating block of glycine receptors reliably led to the emergence of rhythmic motor output that was further enhanced by blockade of GABA_A_ receptors. Output occurred in singlets and multi-burst episodes resembling the intact network. However, burst frequency was slower and individual bursts had longer durations than those produced by the intact preparation. In addition, burst frequency was reduced by noradrenaline and μ-opioids, and increased by serotonin, as observed in the intact network and *in vivo*. These results suggest that the lung area can be activated to produce rhythmic respiratory-related motor output in a reduced brainstem section and provide new insights into respiratory rhythm generation in adult amphibians. First, clustering breaths into episodes can occur within the rhythm-generating network without long-range input from structures such as the pons. Second, local inhibition near, or within, the rhythmogenic center may need to be overridden to express the respiratory rhythm.

## Teaser

**Summary:** We developed a reduced rhythmic preparation from adult bullfrogs that may lead to studies of the cellular mechanisms of respiratory rhythm generation and episode pattern formation in anuran amphibians.

## INTRODUCTION

Breathing is generated by a network of neurons located in the brainstem. Although most work describing the mechanisms of respiratory rhythmogenesis and the organization of the respiratory network has been performed in rodents, amphibians continue to provide insight into the evolution, development and plasticity of breathing (Baghdadwala et al., 2016; Santin, 2019; Milsom et al., 2022; Vasilakos et al., 2004). In mammals, the core of the respiratory network called the preBötzinger Complex (preBötC) is essential for respiratory rhythmogenesis (Smith et al., 1991; Feldman and Kam, 2015). Other rhythmic populations in the network dynamically interact with this core to modulate respiratory output (Smith et al., 2007). Although some have hypothesized that amphibians contain similar types of rhythmic populations for generating lung breathing (Davies et al., 2009; Baghdadwala et al., 2015; Wilson et al., 2002), it remains unknown whether this is the case.

Ambiguity surrounding this issue may have arisen because of inconsistent results across types of pharmacological and transection studies. Amphibians produce two ventilatory behaviors, buccal and lung ventilation, which are thought to be generated by distinct brain stem regions that could, in principle, have rhythmogenic capacity. Buccal ventilation consists of rhythmic airflow into and out of the buccal cavity by reciprocal contraction of the buccal floor depressor and levator muscles. A discrete region located caudal to the vagus nerve spanning rhombomeres 7 and 8 (the ‘buccal area’) is thought to generate buccal ventilation because synaptic blockade near, and microinjection of GABA into, this area inhibits buccal motor patterns (Baghdadwala et al., 2015; Wilson et al., 2002). The other ventilatory behavior, lung ventilation, has two distinct phases, the priming phase and the powerstroke. The priming phase involves buccal floor depression that draws air into the buccal cavity. In the intact animal, this phase is outwardly similar to buccal ventilation, but more recent work suggests that distinct neural networks mediate priming and buccal motor behaviors (Baghdadwala et al., 2015). During the powerstroke phase, compression of the buccal floor rapidly forces the air that entered the buccal cavity during the priming phase into the lungs using positive pressure. This phase is believed to be generated by the ‘lung area’ located in the reticularis parvocellularis (Wilson et al., 2002; Baghdadwala et al., 2015), which is surrounded by the putative ‘priming area’. In addition, a region that encompasses the lung area, potentially overlapping with the priming area, extends broadly through the rostral part of the brainstem and appears to contribute to the rhythmogenic capacity of the powerstroke phase (McLean et al., 1995). Each of these studies demonstrate that certain regions are necessary for rhythmic respiratory motor output. However, they cannot differentiate whether those sites are bona fide rhythm generators or part of a larger circuit that generates rhythmic output via interactions with other structures throughout the brainstem.

If the lung area acts as an endogenous oscillator, it should continue to burst when isolated from other parts of the brainstem. Arguing against the role of the lung area as an endogenous oscillator, transection experiments that isolated this region in a thick slice along with the vagus nerve rootlet do not show respiratory-related motor output in post-metamorphic frogs; specifically, only 2 out of 11 preparations produced any motor activity (Klingler and Hedrick, 2013). In addition, raising the extracellular K^+^ concentration, as commonly done in mammalian preparations, does not reliably initiate the rhythm in a thick slice, with only 3 out of 10 preparations having activity (Klingler and Hedrick, 2013). The influence of high K^+^ likely has a developmental contingency because its elevation increases burst frequency in pre-metamorphic but not post-metamorphic bullfrog brainstems (Winmill and Hedrick, 2003; Klingler and Hedrick, 2013). An alternative reason for the lack of spontaneous rhythmicity in the isolated slice is that unlike the mammalian rhythmic slice preparation, many of the relevant motor neuron cell bodies, the motor nerve rootlet and rhythmic interneurons are not in the same plane, possibly disconnecting the lung area from relevant motoneurons during slicing (Stuesse et al., 1984). In addition to elevated external [K^+^], non-rhythmic mammalian neonatal slices can also be initiated via blockade of inhibitory neurotransmission (Ashhad and Feldman, 2020), suggesting a variety of approaches could be utilized to facilitate bursting in reduced circuits which have not been fully explored in amphibians.

Here, we attempted to determine the conditions, if any, whereby a section of brainstem containing the lung area can reliably generate rhythmic output. For this, we transected the brainstem near the rostral and caudal extent of the vagus nerve root, which formed a thick brainstem slice containing the key elements for a rhythmic motor circuit (motor neuron cell bodies, the motor nerve rootlet and putative rhythmic interneurons). We used a variety of approaches to increase excitability of the network to determine whether this reduced preparation could generate rhythmic output that resembles breathing. To provide evidence that motor bursting produced by this reduced circuit arose from the lung area rhythm generator, we tested the reduced network's sensitivity to neuromodulators with stereotyped actions in the intact network and characterized the episodic output pattern typical of breathing in amphibians.

## MATERIALS AND METHODS

### Animals

All experiments performed were approved by the Institutional Animal Care and Use Committee (IACUC) at the University of Missouri (protocol #39264) and at the University of North Carolina at Greensboro (#19-006 and #2022-1163). Adult female American bullfrogs, *Lithobates catesbeianus* (Shaw 1802) (∼100 g mass; *n*=34), were purchased from Rana Ranch (Twin Falls, ID, USA) and were housed in 20 gallon (∼76 l) plastic tanks containing treated water at 22–25°C bubbled with room air. Frogs had access to wet and dry areas. Frogs were maintained on a 12 h light/dark cycle and fed once per week. Water was cleaned daily for debris and changed weekly.

### Drugs

Strychnine hydrochloride was purchased from Sigma-Aldrich (St Louis, MO, USA). (−)-Bicuculline methiodide, l-glutamate, naloxone hydrochloride and DAMGO were from Hello Bio (Princeton, NJ, USA).

### Brainstem–spinal cord preparation

Brainstem–spinal cord preparations were generated as previously described (Burton and Santin, 2020). Briefly, frogs were deeply anesthetized with isoflurane and decapitated. The head was submerged in ice-cold bullfrog artificial cerebrospinal fluid (aCSF, mmol l^−1^: 104 NaCl, 4 KCl, 1.4 MgCl_2_, 7.5 glucose, 40 NaHCO_3_, 2.5 CaCl_2_ and 1 NaH_2_PO_4_, gassed with 98.5% O_2_, 1.3%CO_2_, pH 7.85) and the forebrain was pithed. The brainstem–spinal cord was then removed, keeping nerve roots intact. Following the dissection, the brainstem was transferred to a chamber superfused with ∼21–23°C aCSF. Cranial nerve X (vagus) activity was recorded using glass suction electrodes immediately following dissection. Recordings were AC amplified (1000×, A–M Systems Model 1700, A–M Systems, Carlsborg, WA, USA), filtered (10 Hz to 5 kHz), and digitized (Powerlab 8/35 ADInstruments, Colorado Springs, CO, USA). Nerve activity was rectified and integrated (time constant=100 ms).

### Experimental protocol

Vagus nerve output from the intact brainstem circuit was recorded for at least 35 min before transection. This output was sampled (‘intact’ in figures) in the last 10 min before transection. Transection was targeted rostral and caudal to the vagal nerve root and was achieved using small spring scissors. Inherent variability in the size of the preparation arose from this method of transection. An experimental workflow showing how brainstems were used throughout experiments is included in Fig. S1.

### Blockade of synaptic inhibition

In a subset of experiments, the antagonist cocktail (1 µmol l^−1^ bicuculline and 3 µmol l^−1^ strychnine) was added to the perfusion shortly following transection (>10 min, *n*=10). Motor output that emerged (9 out of 10 preparations) was allowed to stabilize (>30 min) before neuromodulators were perfused to determine whether the isolated network responded similarly to the intact network. Noradrenaline (norepinephrine; NA) and serotonin (5-HT) were tested together in most preparations (7 out of 9). NA was always tested first; 5 µmol l^−1^ NA was perfused (in the presence of the antagonist cocktail) for 5 min in most preparations, and sampled for a period of 3 min following 1 min of perfusion (7 out of 9). In two preparations where frequency was very slow (<0.5 bursts min^−1^, 2 out of 9), NA was perfused for 10 min instead of 5 min, but the data were nonetheless grouped. Following wash out (>10 min) of NA, a new baseline period of 10 min was established and then 5-HT was perfused in 7 out of 9 preparations tested with NA (in the presence of the antagonist cocktail). Two concentrations of 5-HT were tested for 10 min each with 500 nmol l^−1^ first then 5 µmol l^−1^ 5-HT (7 out of 7). Following 1 min of perfusion, 3 min were sampled. Following the higher dose, 5-HT was then washed out (>10 min) and output was sampled to verify effects were due to 5-HT and not preparation drift. In contrast to prior studies (Belzile et al., 2002; Kinkead et al., 2002), we sampled the dramatic and acute changes in motor output and not steady-state effects that are observed with 5-HT administration because high doses of 5-HT lead to 5-HT receptor desensitization.

In a subset of preparations, 1 µmol l^−1^ bicuculline (*n*=4) or 3 µmol l^−1^ strychnine (*n*=5) was perfused for 1 h shortly following transection (>10 min) to determine the effects of each blocker independently. Following the hour, either 3 µmol l^−1^ strychnine or 1 µmol l^−1^ bicuculline was added to the perfusion to determine the difference between the blocker alone and the antagonist cocktail. In preparations where strychnine was tested alone, following stabilization of output with the antagonist cocktail, DAMGO, a µ-opioid agonist, was perfused for 10 min to test the sensitivity of the reduced network to opioids (*n*=5). Following perfusion of DAMGO, µ-opioid antagonist naloxone (5 µmol l^−1^) was perfused to reverse the effects of DAMGO.

Following facilitation of bursting with the antagonist cocktail, in 5 preparations, the dose of the antagonist cocktail was increased (1 µmol l^−1^ to 5 µmol l^−1^ bicuculline and 3 µmol l^−1^ to 10 µmol l^−1^ strychnine) to determine the dose dependency of motor patterning. This was done in 3 preparations exposed to high K^+^ (described below) and 2 preparations exposed to NA and 5-HT (described above).

### Extracellular K^+^ and glutamate

Extracellular potassium was elevated in 5 preparations to facilitate motor bursting in the non-rhythmic thick slice. Following transection, the preparation was immediately recorded and allowed to recover for >30 min before extracellular potassium was increased from 4 mmol l^−1^ to 7 mmol l^−1^ K^+^ for 1 h (7 mmol l^−1^ K^+^ aCSF; mmol l^−1^: 101 NaCl, 7 KCl, 1.4 MgCl_2_, 7.5 glucose, 40 NaHCO_3_, 2.5 CaCl_2_ and 1 NaH_2_PO_4_, gassed with 98.5% O_2_, 1.3%CO_2_, pH 7.85).

Glutamate was added to the perfusate in 3 preparations to facilitate motor bursting in the non-rhythmic thick slice. Following transection, the preparation was immediately recorded and allowed to recover for >30 min before various concentrations of glutamate (100 nmol l^−1^, 500 nmol l^−1^, 1 µmol l^−1^) were perfused. Each concentration of glutamate was perfused for 10 min in order of increasing concentration, leading to a total exposure time of 30 min.

In high K^+^ (*n*=5) and glutamate (*n*=3), the antagonist cocktail (1 µmol l^−1^ bicuculline and 3 µmol l^−1^ strychnine) was added following washout of these compounds to verify bursting capacity of the isolated network. This gives us confidence that the lack of bursting observed with various manipulations was not due to a lack of ability of the preparation to burst but, rather, a lack of the specific manipulation to facilitate bursting.

In a subset of all transection experiments (*n*=19), size (rostral/caudal axis) of the thick slice was approximated using a caliper with a digital display.

### Intact brainstem experiments

In 6 experiments, cranial nerve V (trigeminal) activity was recorded using glass suction electrodes immediately following dissection. The intact preparations were allowed to stabilize for 1 h before various doses (100 nmol l^−1^, 300 nmol l^−1^, 500 nmol l^−1^, 1 µmol l^−1^, 3 µmol l^−1^, 5 µmol l^−1^) of the µ-opioid agonist DAMGO were perfused for 15 min each in order of increasing concentration to determine the opioid dose that leads to consistent lung burst suppression among preparations. Following elevation to 5 µmol l^−1^ DAMGO, the µ-opioid antagonist naloxone (5 µmol l^−1^) was perfused to reverse the effect of DAMGO. Lung burst frequency was sampled in the last 3 min of each DAMGO dose and for 3 min following full output recovery with naloxone. Buccal frequency was sampled for 1 min total (discontinuous) at baseline and 1 min during 5 µmol l^−1^ DAMGO (continuous).

### Data analysis

Respiratory parameters (burst duration, burst frequency and peak area) were determined from integrated vagus nerve signals using the peak analysis function in LabChart (ADInstruments Inc., Colorado Springs, CO, USA). Singlets and episodes were included in the quantification of burst frequency. Burst start and stop time points were defined as 5% of the height from baseline. For quantification of episodic output, a minimum of 3 episodes were averaged.

### Statistics

**Fig. 1.**
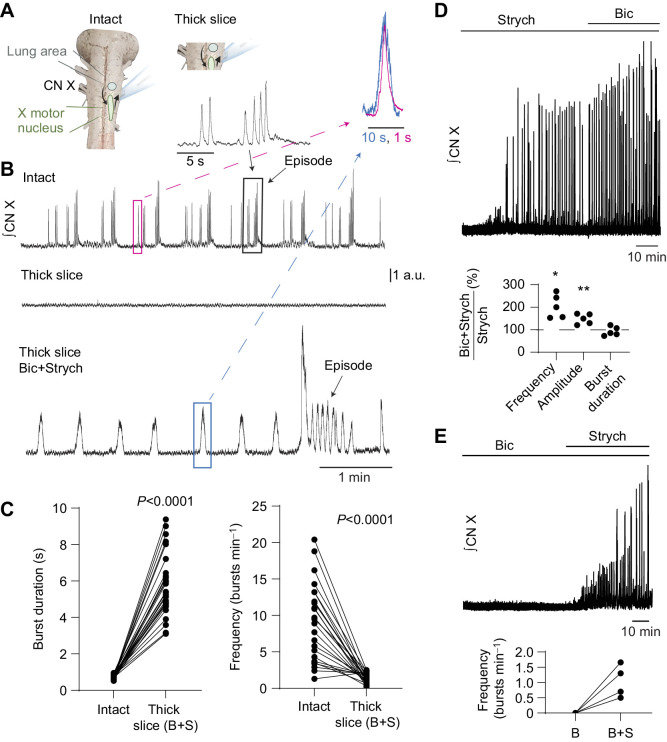
**Blockade of inhibitory neurotransmission promotes rhythmic bursting in thick brainstem sections. (**A) Schematic diagram of the intact brainstem and the transected ‘thick slice’. The ‘lung area’ is thought to generate lung ventilation and activate directly or indirectly relevant motor pools including the vagal motor nucleus. Transection presumably kept part of the lung area and vagal (CN X) motor pool intact. (B) Example traces. Integrated vagus nerve activity (CN X) was rhythmic in the intact brainstem preparation. Transection of the brainstem preparation (thick slice) silenced motor output. In 21 preparations, bath application of antagonist cocktail (1 µmol l^−1^ bicuculline+3 µmol l^−1^ strychnine) led to the emergence of rhythmic motor bursting (B), characterized by significantly increased burst duration (*n*=26) and decreased burst frequency (*n*=25) compared with the intact network (C; *P*<0.0001 by paired *t*-test). Emergence of motor bursting was dependent on glycine receptor block because bath application of 3 µmol l^−1^ strychnine (D), but not 1 µmol l^−1^ bicuculline (E), was able to promote motor bursting alone. However, addition of 1 µmol l^−1^ bicuculline to the perfusion containing 3 µmol l^−1^ strychnine increased the frequency and amplitude but not burst duration of strychnine-induced output (D; *P*<0.05 by one-sample *t*-test). Overlayed and horizontally scaled bursts (pink and blue boxed regions in B) exemplify the similarity in burst shape from the intact network (pink) and thick slice (+ Bic+Strych; blue). The diagram in A was created, in part, using BioRender (biorender.com). a.u., arbitrary units.

Data are presented as means±s.d. A one-sample *t*-test was used to determine whether the mean of samples was statistically different from 100%. When two groups of dependent samples were compared (‘before–after’ experiments), we used a two-tailed paired *t*-test. When three or more groups of dependent samples were compared, we used repeated measures one-way ANOVA and Bonferroni's *post hoc* test. All data were tested for normality using a Kolmogorov–Smirnov test. When non-parametric and comparing three or more groups, a Friedman test was used for one-way repeated measures analysis of variance by ranks. Significance was accepted when *P*<0.05. The ROUT outlier test (*Q*=0.1%) was performed and detected one outlier where burst frequency was unusually high (10 bursts min^−1^) following transection. This experiment was excluded from the frequency data set in Fig. 1C. All analyses were performed using GraphPad Prism (v9.4.1, San Diego, CA, USA).

## RESULTS

To test the hypothesis that a minimal brainstem preparation can generate rhythmic output associated with breathing, we transected the brainstem rostral and caudal to the vagal nerve root (Fig. 1A), generating a slice that was approximately 1.92±0.37 mm thick. We chose these locations, as this would produce a preparation that contains the minimal elements needed for respiratory motor output: the putative lung area, motoneuron pools and a nerve root containing axons that innervate respiratory musculature. In contrast to the intact brainstem, most preparations did not produce any activity (27 out of 28). As this section of the brainstem contains the region thought to give rise to respiratory motor output in amphibians (Wilson et al., 2002), we used a variety of approaches to promote bursting in this non-rhythmic ‘thick slice’, including blockade of synaptic inhibition, elevated extracellular K^+^ and application of glutamate.

### Blockade of synaptic inhibition

**Fig. 2.**
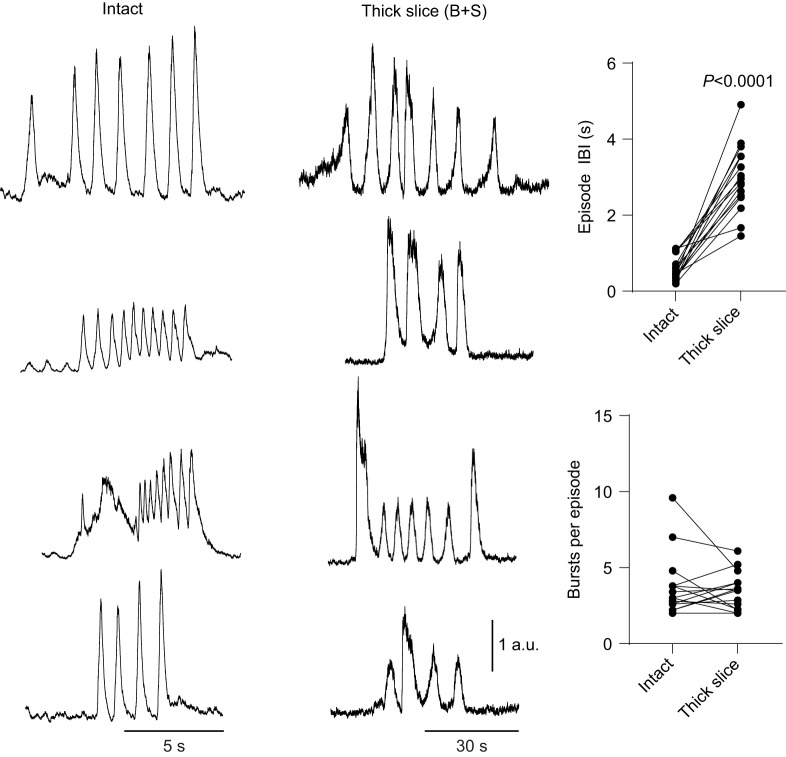
**Comparison of episodic output in intact and reduced networks.** Example paired traces of episodes produced by the intact network and subsequently by the reduced network (thick slice). Episode interburst interval (IBI) was significantly longer in the reduced network (*P*<0.0001 by paired *t*-test), aligning with longer bursts and slowed burst frequency compared with the intact network. However, the number of bursts per episode was not different between preparation types (*P*=0.5197 by paired *t*-test).

In the thick slice, simultaneous blockade of GABA_A_ and glycine receptors (1 µmol l^−1^ bicuculline and 3 µmol l^−1^ strychnine) led to the emergence of persistent bursting in most preparations (21 out of 22 experiments, Fig. 1) within 16.9±13.1 min of exposure to the antagonists. Emergence of motor bursting was dependent on block of glycine receptors but not GABA_A_ receptors because 3 µmol l^−1^ strychnine alone (5 out of 5; Fig. 1D), but not 1 µmol l^−1^ bicuculline alone (4 out of 4; Fig. 1E), promoted motor bursting in the thick slice. However, bicuculline modulated the strychnine-induced activity by increasing the frequency (204.7±52.3% of strychnine alone; *P*=0.011, *n*=5) and amplitude (147.8±22.6% of strychnine alone; *P*=0.0091, *n*=5), but not duration (93.3±20.0% of strychnine alone; *P*=0.4995, *n*=5) of bursts produced by strychnine alone (Fig. 1D). The motor bursts produced by the thick slice in the presence of both bicuculline and strychnine were longer in duration than those in the intact brainstem (Fig. 1C; intact, 0.8±0.1 s; thick slice, 5.9±1.7 s; *P*<0.0001, *n*=26), but had a qualitatively similar shape when scaling and overlaying the two waveforms. Additionally, frequency with the antagonist cocktail in the transected preparation was significantly slower than in the intact network (Fig. 1C; intact, 8.7±4.9 bursts min^−1^; thick slice, 1.0±0.8 bursts min^−1^; *P*<0.0001, *n*=25). Interestingly, most rhythmic preparations (24 out of 26) exhibited clustered bursting activity similar to the episodic patterning observed in the intact brainstem (Kinkead et al., 1994) (Fig. 2). In preparations that had episodes both before and following transection (16/24), the number of bursts per episode was not different between the intact preparation and thick slice (Fig. 2; intact, 3.6±2.0 bursts episode^−1^; thick slice, 3.4±1.2 bursts episode^−1^; *P*=0.5197, *n*=16). However, the interburst interval during episodes was much longer in the thick slice (Fig. 2; intact, 0.59±0.31 s; thick slice, 2.97±0.87 s; *P*<0.0001, *n*=16), aligning with the decreased frequency and increased burst duration we observed in the reduced circuit compared with the intact network.

**Fig. 3.**
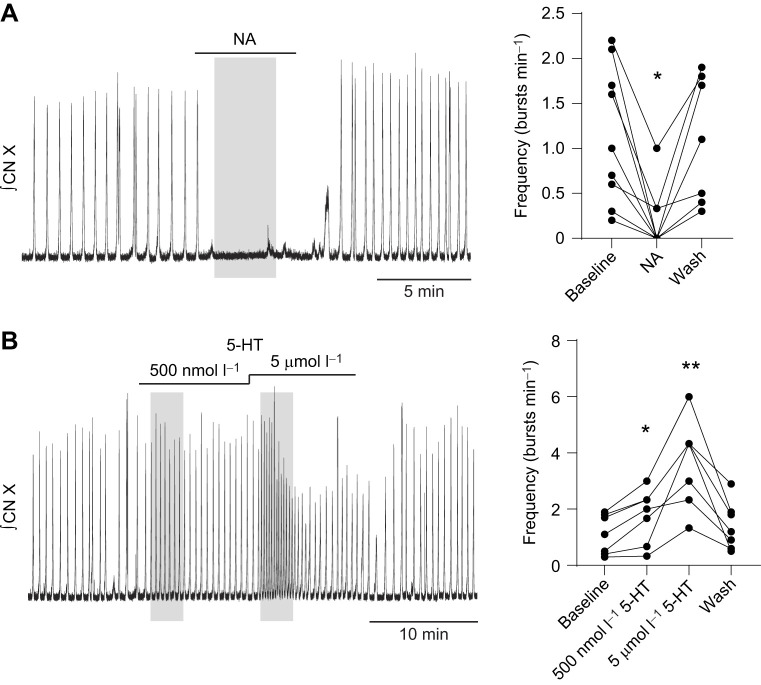
**Neuromodulators reliably change burst frequency in the thick slice preparation.** Integrated vagus nerve activity (CN X) was rhythmic in the thick slice that was perfused with antagonist cocktail (1 µmol l^−1^ bicuculline+3 µmol l^−1^ strychnine; baseline). (A) Bath application of 5 µmol l^−1^ noradrenaline (NA) significantly decreased burst frequency (*P=0.0029, *n*=9) compared with baseline. (B) Bath application of 500 nmol l^−1^ and 5 µmol l^−1^ serotonin (5-HT) significantly increased burst frequency (*P=0.0360, **P=0.0072, respectively, *n*=7) compared with baseline. Burst frequency following washout was not significantly different from baseline. NA treatment was compared with baseline by Friedman's test and Dunn's *post hoc* test. 5-HT treatment was compared with baseline by repeated measures one-way ANOVA and Bonferroni's *post hoc* test.

The above results demonstrate that the thick slice can produce rhythmic output that also occurs in episodes. To determine whether the reduced network producing this motor output had additional properties comparable to those of the intact circuit, we tested the effects of neuromodulators that have been previously described in the intact brainstem from adult frogs. NA is well known to reduce lung burst frequency, while 5-HT increases lung burst frequency (Adams et al., 2021; Belzile et al., 2002; Fournier and Kinkead, 2006; Kinkead et al., 2002). Thus, we rationalized that if both modulators similarly influence bursting in the thick slice, then activity might arise from the same set of rhythmic neurons. Bath application of NA to the thick slice caused a rapid and significant reduction in burst frequency, often eliminating bursting (Fig. 3A; baseline, 1.2±0.8 bursts min^−1^; NA, 0.2±0.3 bursts min^−1^; *P*=0.0029, *n*=9). Bath application of low-dose 5-HT (500 nmol l^−1^) to the thick slice rapidly and significantly increased motor burst frequency (Fig. 3B; baseline, 1.1±0.7 bursts min^−1^; 500 nmol l^−1^ 5-HT, 1.8±0.9 bursts min^−1^; *P*=0.0360, *n*=7). Subsequent bath application of high-dose 5-HT (5 µmol l^−1^) led to a more drastic, significant increase in motor burst frequency (Fig. 3B; 5 µmol l^−1^ 5-HT, 3.6±1.5 bursts min^−1^; *P*=0.0072, *n*=7) that often decayed during the 10 min exposure, potentially due to receptor desensitization (Yao et al., 2010).

**Fig. 4.**
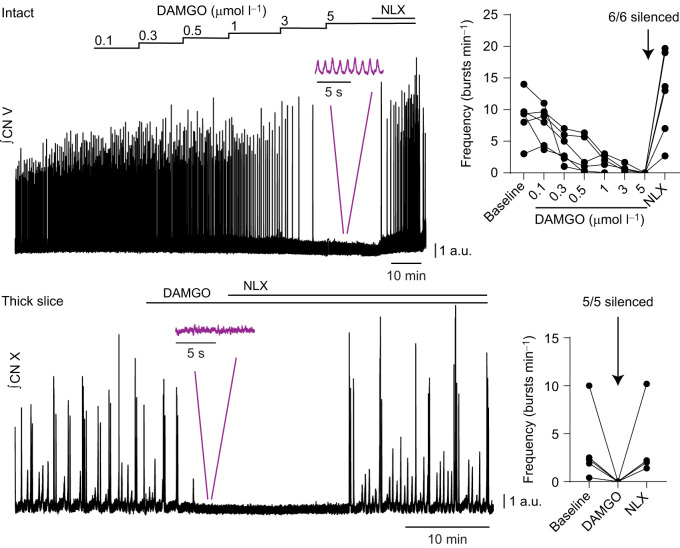
**Lung bursts in the intact network and motor bursts produced by the reduced network are silenced by µ-opioids.** Integrated trigeminal nerve activity (CN V) was recorded in the intact brainstem preparation and integrated vagus nerve activity (CN X) was recorded in the thick slice. A representative trace of rhythmic motor output suppression during application of increasing concentrations of the µ-opioid agonist DAMGO is shown on the left for both preparations. Output from all intact brainstem preparations was silent during 5 µmol l^−1^ DAMGO application (top right). However, in 3 out of 3 preparations where buccal activity was detectable on the trigeminal nerve, buccal activity persisted in 5 µmol l^−1^ DAMGO (inset, purple). Bursting emerged from quiescence following perfusion of the µ-opioid antagonist naloxone (NLX, 5 µmol l^−1^). As for the intact network, output produced by inhibition block in the thick slice was silenced by 5 µmol l^−1^ DAMGO and subsequently restored with naloxone in all preparations (bottom right).

In addition to NA and 5-HT, μ-opioids present an interesting test of the lung area. The μ-opioid agonist DAMGO selectively depresses lung motor output without affecting the ability of the buccal area to generate a rhythm (Vasilakos et al., 2004). To determine whether the output produced by the reduced network was consistent with the lung area in the intact network, we tested whether DAMGO depressed activity in the thick slice. We first determined the opioid dose needed to suppress lung output in the intact network. Although DAMGO inhibited lung output over a range of doses among intact preparations (Fig. 4), most preparations (4 out of 6) were not fully suppressed until 5 µmol l^−1^. Additionally, in 3 intact preparations where buccal activity was prominent on the trigeminal nerve (Fig. 4, inset), we observed a persistence of buccal activity during application of 5 µmol l^−1^ DAMGO (baseline, 55.0±13.2 bursts min^−1^; 5 µmol l^−1^ DAMGO, 65.0±19.1 bursts min^−1^; *n*=3), aligning with the differential sensitivity of lung and buccal activity to µ-opioids previously reported (Vasilakos et al., 2004). To test the opioid sensitivity of the thick slice, we bath-applied 5 µmol l^−1^ DAMGO because it led to consistent silencing of lung but not buccal activity in the intact network. Indeed, output from 5 out of 5 reduced preparations was silenced by DAMGO (Fig. 4), aligning with results from lung activity, but not buccal activity, in the intact preparation. Opioid-mediated output suppression in all preparations was reversed with the μ-opioid antagonist naloxone. Altogether, motor output produced by the thick slice has a similar profile of modulatory sensitivity to motor output associated with lung breathing that is produced by the intact circuit.

**Fig. 5.**
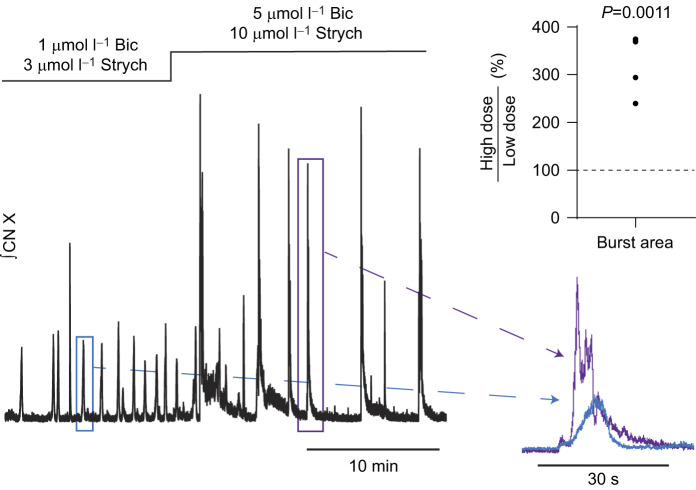
**Increasing the dose of antagonist cocktail transforms motor pattern in the thick slice preparation.** Integrated vagus nerve activity (CN X) was recorded in the thick slice. Increasing the concentration of antagonist cocktail from 1 µmol l^−1^ bicuculline + 3 µmol l^−1^ strychnine to 5 µmol l^−1^ bicuculline + 10 µmol l^−1^ strychnine led to significant increases in motor burst area (right; normalized to 1 µmol l^−1^ bicuculline+3 µmol l^−1^ strychnine), characterized by large motor bursts with decrementing shape in 5 out of 5 preparations. Overlayed bursts exemplify the difference in output from the low dose (inset, blue) of antagonist cocktail to the high dose (inset, purple; *P*=0.0011 by one-sample *t*-test).

To determine the dose dependency of the motor output pattern, the concentration of antagonist cocktail was increased to 5 µmol l^−1^ bicuculline and 10 µmol l^−1^ strychnine, which has been suggested to elicit lung-like activity in isolated parts of the respiratory network (Reed, 2017). Increasing the dose of antagonist cocktail transformed motor output to predominately large bursts with decrementing shape (Fig. 5) that were unlike the dominant pattern of motor bursts produced at lower concentrations (Fig. 5, inset). These large bursts had significantly increased area compared with the bursts produced by lower doses of antagonist cocktail (329.4±60.4% of low-dose burst area; *P*=0.0011, *n*=5; Fig. 5). Overall, these experiments show that 1 µmol l^−1^ bicuculline and 3 µmol l^−1^ strychnine appear to selectively activate motor output driven by the lung area in a reduced preparation.

### Extracellular K^+^ and glutamate

**Fig. 6.**
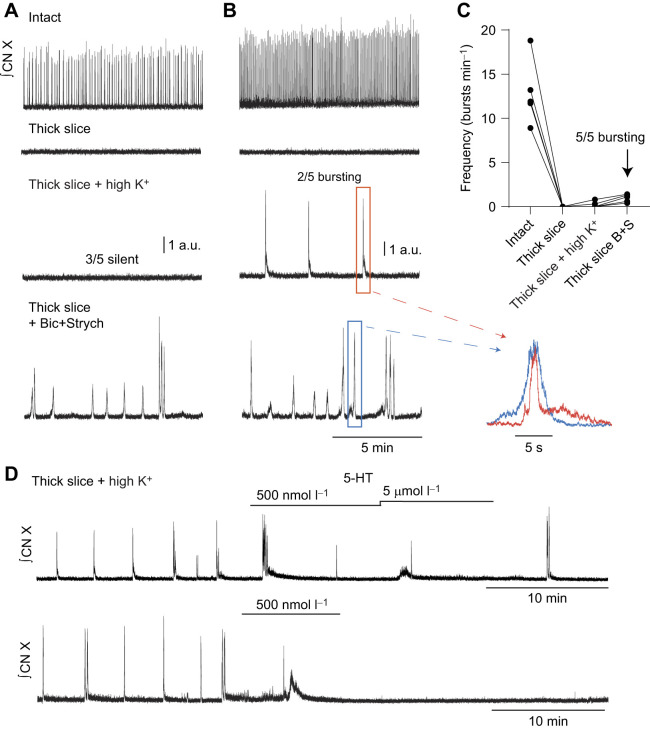
**Elevation of extracellular potassium does not reliably elicit bursting in the thick slice preparation.** (A,B) Integrated vagus nerve activity (CN X) was rhythmic in both intact brainstem preparations. Transection of the brainstem preparation silenced motor output (thick slice). Elevation of extracellular potassium (7 mmol l^−1^, ‘high K^+^’) failed to produce rhythmic bursting in 3 out of 5 thick slice preparations (A, example trace) but led to the emergence of bursting in 2 out of 5 preparations (B, example trace). After washout, bath application of antagonist cocktail (1 µmol l^−1^ bicuculline+3 µmol l^−1^ strychnine) led to persistent bursting in 5 out of 5 thick slice preparations. Motor bursts in elevated potassium (inset, orange) had a qualitatively different shape from bursts in antagonist cocktail (inset, blue). The lack of bursting observed with elevated potassium in 3 out of 5 preparations was not due to the inability of the preparation to burst but rather to the inability of elevated potassium to reliably facilitate bursting. (C) Summary of results. (D) Example traces of both rhythmic preparations that were initiated by elevation of extracellular potassium and then exposed to 5-HT.

Block of synaptic inhibition would serve to enhance excitability within the slice. To determine whether activation of the motor pattern was specific to block of synaptic inhibition versus a generalized response caused by enhanced excitability, we tested additional treatments that increase excitability in the thick slice. In the neonatal rodent slice preparation, elevated external K^+^ is often used to sustain rhythmic output (Johnson et al., 2001; Tryba et al., 2003; Kam et al., 2013). In 3 out of 5 preparations increasing extracellular potassium failed to initiate rhythmic vagal motor bursting (Fig. 6). In contrast, in 2 preparations, increased extracellular K^+^ led to the emergence of motor bursts (Fig. 6). However, bursts in these two preparations were suppressed, rather than enhanced, by 5-HT and had a qualitatively different shape compared with bursts elicited by bicuculline/strychnine (Fig. 6). Interestingly, in one of the two preparations, bursting ceased during application of 500 nmol l^−1^ 5-HT and did not recover following washout of 5-HT. After washout of aCSF containing high K^+^, blockers of synaptic inhibition were bath applied (1 µmol l^−1^ bicuculline+3 µmol l^−1^ strychnine), which promoted bursting in 5 out of 5 preparations exposed to high K^+^. Taken together, high K^+^ does not mimic the effects of blocking synaptic inhibition with 1 µmol l^−1^ bicuculline and 3 µmol l^−1^ strychnine.

**Fig. 7.**
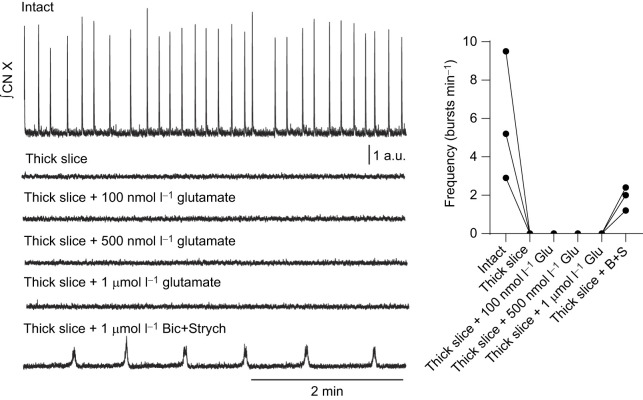
**Perfusion of glutamate, at various doses, does not elicit bursting in the thick slice preparation.** Integrated vagus nerve activity (CN X) was rhythmic in the intact brainstem preparation. Transection of the brainstem preparation (thick slice) silenced motor output. Perfusion of 100 nmol l^−1^ glutamate failed to produce rhythmic bursting in 3 out of 3 preparations. Further elevation of glutamate concentration (500 nmol l^−1^ or 1 µmol l^−1^ glutamate) also failed to facilitate motor bursting. In contrast, bath application of antagonist cocktail (1 µmol l^−1^ bicuculline+3 µmol l^−1^ strychnine) facilitated rhythmic motor bursting. Thus, the lack of bursting observed with glutamate perfusion was not due to the inability of the preparation to burst but rather to the inability of glutamate to facilitate bursting.

Finally, rhythmic bursting and pattern formation in the mammalian preBötC may involve activation of AMPA, NMDA and metabotropic glutamate receptors (Mironov, 2008; Pace and Del Negro, 2008; Funk, 2013). Thus, we also applied various concentrations of glutamate to the preparation to determine whether this treatment could elicit bursting. Bath application of glutamate at 100 nmol l^−1^, 500 nmol l^−1^ and 1 µmol l^−1^ failed to elicit bursting in all preparations tested (3 out of 3; Fig. 7). Taken together, these results suggest that subsaturating block of synaptic inhibition dose-dependently initiates motor patterns consistent with lung ventilation in a reduced slice preparation and does not appear to be a generalized response to enhanced excitability in the slice.

## DISCUSSION

Here, we tested the hypothesis that a reduced section of the frog brainstem that contains the lung area can produce rhythmic output consistent with the respiratory network. Although this thick slice contains the minimal circuit requirements for breathing, it was not rhythmic under standard conditions, as previously shown by Klingler and Hedrick (2013). However, using several pharmacological approaches, we show that low-dose blockade of synaptic inhibition initiates motor bursting that recapitulates salient features of the respiratory motor output from more intact preparations.

### Comparison with the intact network

We show that subsaturating doses of bicuculline and strychnine produce rhythmic bursting in the thick slice (Fig. 1A). Although the scaled burst shape was similar between the intact and thick slice preparations, burst duration was increased and burst frequency was strongly decreased compared with the intact brainstem. Thus, the obvious question arises: is this activity produced by the respiratory network in the adult bullfrog? Below, we detail our interpretation as to why we believe this activity is respiratory related and discuss implications for the understanding of respiratory rhythm generation and pattern formation in anuran amphibians.

First, studies on the control of breathing are frequently done using reduced preparations, where motor output associated with breathing (Suzue, 1984; Smith et al., 1991; Saunders and Levitt, 2020) is longer and less frequent than *in vivo (*Walker et al., 1997; Zehendner et al., 2013). We speculate that changes observed in burst duration and frequency potentially arose from removal of populations of neurons that project to the rhythm generator, which normally serve to modulate respiratory rate and breath duration. Indeed, removal of rostral midbrain or the dorsal portion of the caudal medulla slows respiratory frequency and increases burst duration (Gargaglioni et al., 2007; Baertsch et al., 2019; Amaral-Silva and Santin, 2022). Next, although subsaturating blockade of glycinergic inhibition was required to rescue the rhythm in the slice (Fig. 1), we suggest that this manipulation may counterintuitively act to reduce frequency of this preparation relative to the intact network. Interestingly, in more intact preparations, pharmacological blockade of either glycinergic or GABAergic inhibition, albeit at higher doses than used in our low-dose antagonist cocktail, increases burst duration and decreases frequency (Broch et al., 2002), similar to values we report for the thick slice. In addition, the block of inhibition in the mammalian preBötC and neighboring BötC *in vivo* leads to increased burst duration and decreased burst frequency that is, to some extent, potentiated by subsequent vagotomy (Janczewski et al., 2013). Thus, although blockade of glycine receptors was required to initiate activity in the thick slice, this manipulation may set the network at lower frequency with broader bursts. Taken together, the increased duration and decreased frequency of bursts compared with the intact respiratory network may result from the loss of neurons that project to the lung area that modulate bursting and/or pharmacological block of glycinergic inhibition required to initiate activity in this preparation.

Second, only low-dose block of inhibitory synaptic transmission led to respiratory-like bursting, while higher doses led to output consistent with ‘seizure-like’ activity. The dose of strychnine used in our experiments (3 µmol l^−1^) is within the range of doses (2.5–5 µmol l^−1^) that have been shown to preserve motor patterning of breathing in the bullfrog brainstem preparation (Kimura et al., 1997; Broch et al., 2002), while higher doses of strychnine (10–25 µmol l^−1^) result in large decrementing bursts (Kimura et al., 1997; Broch et al., 2002) that resemble seizure-like activity observed in other vertebrate preparations (Mahrous and Elbasiouny, 2017; Cohen and Harris-Warrick, 1984). In accordance with prior literature, the bursts we observed with 10 µmol l^−1^ strychnine appear strikingly similar to the bursts produced by the intact network at the same dose (Kimura et al., 1997; Broch et al., 2002), further suggesting bursts we observed at the low dose were not some manifestation of massive network synchrony. Likewise, in the 2 out of 5 individual experiments where raising extracellular K^+^ induced activity, bursts occurred sporadically, had a different qualitative shape, and responded differently to 5-HT compared with bursts that occurred with lower doses of bicuculine/strychnine. Furthermore, the bursting we observed that was induced by increased extracellular K^+^ qualitatively corroborates the experiments by Klingler and Hedrick (2013) showing that an isolated section of the brainstem from post-metamorphic bullfrogs containing the vagus nerve root is largely quiescent in normal conditions and unreliably activated by elevated potassium. Taken together, when bursts were generated from the thick slice with elevated potassium or high doses of antagonist cocktail, they seemed to arise through different mechanisms from respiratory bursts that were activated by low-dose bicuculine and strychnine.

Finally, the sensitivity of this output to neuromodulators matched known responses in the intact brainstem. In reduced mammalian preparations, changes in frequency in response to application of neuromodulators, 5-HT and NA, occur as a result of actions within rhythm-generating populations (Al-Zubaidy et al., 1996). Accordingly, we hypothesized output from the thick slice would be modulated by NA and 5-HT in a similar way to the intact network. Application of NA consistently suppressed motor output in the thick slice in accordance with effects of NA observed in the intact circuit (Fournier and Kinkead, 2006; Adams et al., 2021), while application of 5-HT consistently increased motor burst frequency in the thick slice (Belzile et al., 2002; Kinkead et al., 2002). Previous work has shown that bullfrog lung output is sensitive to µ-opioids (Vasilakos et al., 2004; Davies et al., 2009). Indeed, we found lung bursting in the intact network and motor bursting in the reduced network were silenced by DAMGO, a µ-opioid agonist, further supporting the idea that bursting in the thick slice was respiratory related. It is also worth mentioning that our preparations may have contained both the region involved in buccal ventilation and part of the priming area. However, the buccal rhythm is not similarly sensitive to NA (Adams et al., 2021), nanomolar doses of 5-HT (Kinkead et al., 2002) and µ-opioids (Vasilakos et al., 2004), strongly suggesting this activity was not caused by the buccal area. Regarding the putative priming area, in the intact network, the vagus nerve does not appear to receive significant priming input and has not been shown to burst independently from the lung area (Baghdadwala et al., 2015). Thus, it is unlikely that the activity we observed resulted from the priming area alone. However, it is possible that disinhibition in the thick slice could lead to an unmasking of priming activity onto the vagal motor pool, which is not normally present, causing it to occur along with the lung output. Taken together, and despite differences compared with the intact central respiratory network, these experiments suggest that we activated a key microcircuit involved in amphibian lung breathing in a reduced slice preparation.

### How does inhibition block induce respiratory bursts?

An interesting aspect of these results is that blockade of synaptic inhibition did not immediately restart rhythmic output, and other manipulations that enhance excitability did not initiate activity. The initiation of bursting was due to block of glycinergic, and not GABAergic, receptors because strychnine alone and not bicuculline alone promoted motor bursting in the thick slice. Therefore, a generalized increase in network excitability does not seem to explain why the block of glycine receptors restarted the network. Given that modulatory compounds such as NA and 5-HT alter output of the network rapidly, this delay and lack of evidence for general enhancement of excitability to restore activity suggests that the block of glycinergic inhibition acts through a more complex mechanism than straightforward removal of tonic inhibition. We suggest that plasticity induced by the block of glycinergic inhibition may restore rhythmic output. Indeed, block of inhibitory synaptic receptors can induce synaptic plasticity that occurs through signaling processes directly through receptor inhibition, rather than through changes in network activity or excitability (Lévi et al., 1998; Garcia-Bereguiain et al., 2016; Gonzalez-Islas et al., 2018). Through this lens, the rostral boundary of the transected brainstem was in the range of rhombomeric segments 5 and 6, where the lung area has been proposed to be located (Baghdadwala et al., 2015), suggesting part of the lung area may have been removed in our reduced preparation (Fig. 1A). Thus, the ‘zone of active neurons’ could have shifted as a result of strychnine-induced plasticity. An alternative possibility is that transection removes critical vagal motor neurons in the caudal part of the motor pool that contributes to motor output in the intact circuit (Amaral-Silva and Santin, 2022). In the several minutes following block of synaptic inhibition, we suspect that reserve motor neurons may be recruited to express the respiratory rhythm in this preparation through rapid plasticity. Altogether, our results align with the idea that the distribution of rhythmic respiratory neuron activity is dynamic, and inhibition itself modulates the extent of rhythmically active neurons in the ventral respiratory column (Baertsch et al., 2019).

### Implications for episode formation in anuran amphibians

One surprising result from these experiments was that almost all rhythmic slices exhibited burst episodes that resemble those of the intact brainstem (Fig. 2) and episodic breathing *in vivo* (Santin and Hartzler, 2016). Episodic breathing in diverse vertebrate species is thought to rely on pontine structures that provide descending input to medullary respiratory regions (Milsom et al., 1997). Indeed, early transection experiments that separated the optic lobes and cerebellum from the brainstem suggest midbrain centers play a critical role in episode formation in bullfrogs (Oka, 1958). These results were corroborated by Kinkead et al. (1997) and Gargaglioni et al., (2007), who produced evidence that the nucleus isthmus (NI), a midbrain structure, provides tonic drive to an unknown respiratory center(s) to promote episodes. However, more recent evidence suggests episode formation is an inherent property of medullary rhythm-generating circuits (Fong et al., 2009) that is partly dependent on GABA- and glycine-mediated processes (Straus et al., 2000; Vasilakos et al., 2006; Fong et al., 2009). Specifically, inhibitory neurotransmission is required (Vasilakos et al., 2006), but too much suppresses episode formation (Straus et al., 2000), suggesting a proper balance of inhibition is needed to generate episodes. Corroborating the role of inhibition in episode formation, we observed episodic output in most reduced rhythmic preparations that had been initiated by subsaturating block of synaptic inhibition, which lacked the pons and other relevant respiratory structures (Fig. 1A). Thus, these results provide evidence that the respiratory rhythm-generating network may be inherently episodic.

How do we reconcile these results with seemingly clear data demonstrating a role of midbrain structures in episodic breathing in anuran amphibians? Given that subsaturating block of inhibitory neurotransmission produced episodic output in the thick slice, we speculate that the expression of episodes *in vivo* may require the local inhibition to be overridden by input from the midbrain. Thus, block of inhibitory neurotransmission in the thick slice may normally be achieved through the NI *in vivo*. Additionally, we cannot definitively rule out the existence of multiple rhythm-generating populations in the slice. It is possible that the thick slice contained part of the buccal, priming and lung areas based on their proposed locations (Baghdadwala et al., 2015). Perhaps the episodic output we observed was due to interactions between the buccal and lung oscillator as hypothesized previously (Bose et al., 2005; Vasilakos et al., 2006). Another possibility is that the putative priming area inhibits the lung area, and this inhibition needs to be overridden to express episodes (Baghdadwala et al., 2015). In either case, episodes have been shown to occur in a quantal pattern (Vasilakos et al., 2004; Fong et al., 2009), which supports the idea that episode formation is a manifestation of coupled oscillators (Mellen et al., 2003). The relationship between single or multiple brainstem regions that permit breathing episodes remains to be elucidated, but our data strongly suggest this behavior can arise locally without long-range input.

### Conclusion

In sum, we found that subsaturating block of inhibitory neurotransmission led to the emergence of motor bursting in a thick section of medullary tissue that is consistent with breathing. Output from this reduced network was slower but behaved similarly to the intact bullfrog respiratory network in terms of motor output shape, episode formation and responsiveness to different neuromodulators. Given the utility of amphibians in addressing questions that relate to the evolution and development of air breathing, we propose that the thick slice preparation may provide a way to study mechanisms of respiratory rhythm generation and episode pattern formation to inform these critical aspects of vertebrate physiology.

## Supplementary Material

10.1242/jexbio.245951_sup1 Supplementary information

## ECR Spotlight

This article has an associated ECR Spotlight interview with Sandy E. Saunders.
