# Supplementary material for: Activation of respiratory-related bursting in an isolated medullary section from adult bullfrogs
Source: J Exp Biol. 2023 Sep 22;226(18):jeb245951. doi: 10.1242/jeb.245951 (PMC10546875; doi:10.1242/jeb.245951)
Supplement: Supplementary information [file jexbio-226-245951-s1.pdf]

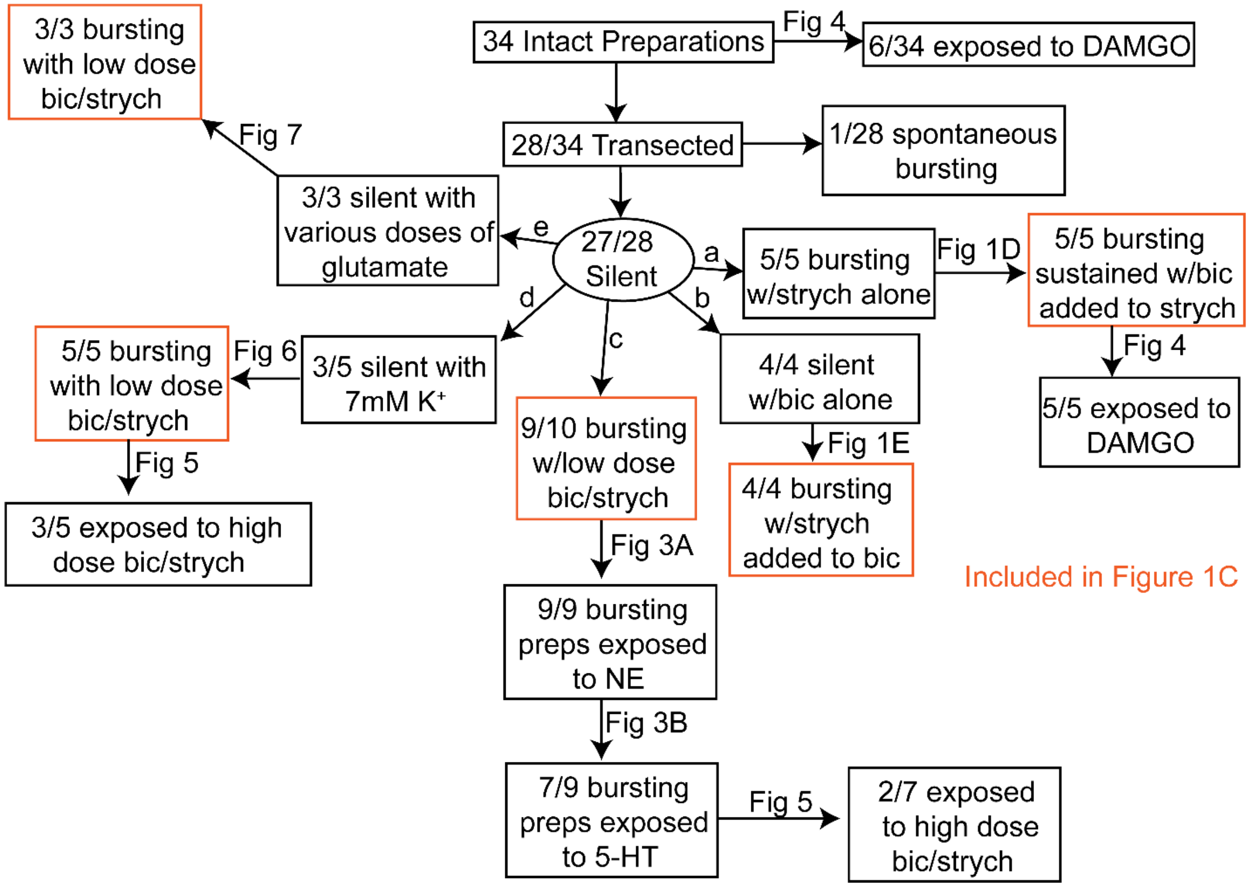

Fig. S1. Experimental Workflow

34 brainstems were dissected and recorded (top middle). 6 of those 34 brainstems were exposed to µ opioid agonist DAMGO (top left). 28 out of 34 intact preparations were transected rostral and caudal to the vagus nerve root. 27 out of 28 reduced preparations were silent. 1 out of 28 reduced preparations produced spontaneous bursts. a) Addition of 3 µM strychnine (strych) to the reduced preparation produced bursting in 5 out of 5 preparations (5 out of 27 silent). Addition of 1 µM bicuculline (bic) to 3 µM strych sustained but modulated bursting. Following stabilization, 5 out of 5 preparations were exposed to DAMGO. b) Addition of 1 µM bic did not produced bursting in 4 out of 4 preparations (4 out of 27 silent). Addition of 3 µM strych to 1 µM bic promoted bursting in 4 out of 4 preparations. C) Addition of 1 µM bic and 3 µM strych produced bursting in 9 out of 10 preparations (10 out of 27 silent). 9 out of 9 rhythmic preparations were exposed to norepinephrine (NE). 7 out of the 9 rhythmic preparations exposed to NE

Journal of Experimental Biology • Supplementary information

were exposed to 5-HT. 2 of those 7 preparations exposed to 5-HT were exposed to high dose bic/strych (5 µM bic and 10 µM strych). d) Elevation of extracellular potassium produced bursting in 2 out of 5 preparations (5 out of 27 silent). Following washout of elevated potassium, addition of 1 µM bic and 3 µM promoted bursting in 5 out of 5 preparations. 3 out of those 5 preparations were exposed to high dose bic/strych (5 µM bic and 10 µM strych). e) Addition of various doses of glutamate to the reduced preparation did not produce bursting in 3 out of 3 preparations. Addition of 1 µM bic and 3 µM strych promoted bursting in 3 out of 3 preparations. All experiments where 1 µM bic and 3 µM strych were perfused together in the reduced network were sampled and pooled for data in Figure 1C (orange outline).

Journal of Experimental Biology • Supplementary information
